# Spherical Silica Modified with Magnesium and Ruthenium—Synthesis, Characterization and Catalytic Properties

**DOI:** 10.3390/ma14237378

**Published:** 2021-12-02

**Authors:** Kalina Grzelak, Maciej Trejda

**Affiliations:** Department of Heterogeneous Catalysis, Faculty of Chemistry, Adam Mickiewicz University, Uniwersytetu Poznańskiego 8, 61-614 Poznań, Poland; tmaciej@amu.edu.pl

**Keywords:** nanospheres, mesoporous silica, ruthenium species, magnesium species, bimetallic catalyst, hydrogenation

## Abstract

The design of different bimetallic catalysts is an important area of catalytic research in the context of their possible applications in the cascade processes, meeting the requirements of the so-called green chemistry. In this study, such catalysts were obtained by the incorporation of magnesium species into spherical silica, which was in the next step covered with porous silica and modified with ruthenium species. The structure and chemical composition of the materials obtained were determined by XRD measurements, low temperature N_2_ adsorption/desorption, SEM, ICP-OES and XPS methods. The catalytic activities of materials obtained were tested in 2-propanol decomposition and hydrogenation of levulinic acid. The results obtained confirmed the successful coverage of nanospheres with porous silica. A much higher concentration of ruthenium species was found on the surface of the catalysts than in their bulk. The opposite relationship was observed for magnesium species. The modification of nanospheres with silica had a positive effect on the catalytic activity of the materials obtained. For the most active sample, i.e., Ru/NS/3Mg/NS, 49% of levulinic acid conversion in its hydrogenation process was reported with γ-valerolactone as the only product.

## 1. Introduction

Porous materials play a crucial role in heterogeneous catalysis as they facilitate diffusion of the reactants and dispersion of the active phase. These properties are much desired when designing a catalytic support. One of the most common oxides used for porosity generation is silica. Various structures of silica with large surface areas are well documented, i.e.,: MCM-41 [1], SBA-15 [2], MCF [3], KIT-6 [4] and many of their derivatives. Over the years spherical silica has been paid much attention, as it can be applied as a hard template [5,6], a hollow sphere capturing a metal inside, or as a traditional matrix. Selective generation of different types of pores has endowed the silica with interesting properties. For instance Niu et al. [7] have proposed the synthesis of core–shell-structured silica spheres with large (~14 nm) pores in the core and small pores (2 nm) in the shell with the use of various surfactants. Other authors have employed silica with core–shell structure to separate basic (APTMS) and acidic (MPTMS) active sites [8]. Such layered systems can be also applied for encapsulation of metal nanoparticles to prevent their aggregation [9]. Selective segregation of the active phase and encapsulation can prevent its potential leaching, especially in liquid phase reactions. An exemplary process is biomass transformation into fine chemicals that is usually carried out in a liquid medium. In such reactions the application of a heterogeneous catalyst would permit easy separation of the catalyst and would eliminate the step of the post-reaction medium neutralization.

Levulinic acid (LA) is one of the platform molecules derived from biomass (such as lignin, cellulose). When hydrogenated, it is transformed to γ-valerolactone (GVL), which can be applied as fuel, food additive, solvent and as a precursor in the synthesis of fine chemicals [10]. The production of GVL usually requires high temperature and elevated pressure. Oxides with diverse properties have been tested in the hydrogenation of LA: bases (ZnO, MgO) and acids (ZrO_2_, Al_2_O_3_, TiO_2_) [11]. The use of a base such as MgO has been reported to improve the yield of the desired product and to increase the GVL formation rate [12]. Additionally, it has also been postulated that MgO could activate the carbonyl group of levulinic acid [13]. Acidic sites on the other hand are important in the last step of dehydration and cyclization [11]. As for monometallic heterogeneous catalysts explored in the reaction, noble metals such as Pd, Pt, Ru, Rh have been involved in catalyst development with Ru/C, reportedly exhibiting the highest yield to GVL [14,15,16]. There have been extensive studies concerning the use of non-noble metals, however the noble metals based systems are still more efficient [11]. Recently, ruthenium loaded on MgO was reported to be a very attractive catalyst in another hydrogenation reaction of aromatic benzyltoluenes [17]. Ruthenium nanoparticles embedded in silica nanospheres found application in hydrolytic dehydrogenation of ammonia borane with much lower activation energy for the hydrolysis than Ru/C proving structure-wise effects [18].

In this work, we proposed the application of a catalyst based on ruthenium and magnesium loaded onto silica, in liquid phase hydrogenation of levulinic acid to GVL. The idea of this work was to localize magnesium species in the pores of spherical silica, which in the next step was modified with porous SiO_2_ and finally modified with ruthenium.

## 2. Materials and Methods

### 2.1. Materials

- Cetyltrimethylammonium bromide (CTAB), >98% (Aldrich, St. Louis, MO, USA);
- Tetraethoxysilane (TEOS), >99% (Aldrich, St. Louis, MO, USA);
- Na_2_CO_3_, 99.5% (Aldrich, St. Louis, MO, USA);
- Ethanol, 96% (Stanlab, Lublin, Poland);
- Magnesium acetate tetrahydrate, >99% (Aldrich, St. Louis, MO, USA);
- Ruthenium trichloride hydrate, 35%–40% of Ru (Acros Organics, Geel, Belgium);
- 2-propanol (Stanlab, Lublin, Poland);
- Levulinic acid, >99% (Aldrich, St. Louis, MO, USA).

### 2.2. Synthesis of the Support

Nonporous silica spheres were prepared according to the procedure described in [19]. Ethanol (243 mL) and ammonia (82 mL) were mixed together and heated up to 30 °C. Then, 11.76 g of TEOS were added at once under vigorous stirring (1000 rpm). After 1 h the as-obtained white solid was filtered off by centrifugation (5000 rpm), washed with water and ethanol and moved to an oven to dry overnight at 50 °C in air in steady-state conditions. The synthesis was repeated a few times and the obtained solids were mixed. Next, silica was submitted to the etching process, based on the procedure reported in [20]. For this purpose, 4.5 g of the synthesized powder was dispersed in 810 mL of water for 15 min using ultrasounds. In the next step, 90 mL of CTAB solution (12.5 mg·mL^−1^) was added. After stirring the solution for 30 min, 19.08 g of powder Na_2_CO_3_ was added, and the stirring was continued for 24 h at 35 °C. The precipitated powder was centrifuged (4500 rpm) and washed with 100 mL of water 5 times. Finally, the obtained material was dried at room temperature and calcined at 550 °C for 6 h (temperature ramp 1.5 °C·min^−1^). The obtained silica nanospheres were denoted with “NS” acronym.

### 2.3. Modification with Magnesium

The silica matrix was impregnated (wetness impregnation) with three different amounts of magnesium, using aqueous solution of magnesium acetate tetrahydrate (2.5 mL of magnesium precursor aqueous solutions of concentration 2.17, 4.57, 7.26 mmol/ g of SiO_2_). The samples were dried at 80 °C and calcined at 500 °C for 5 h (temperature ramp 1 °C·min^−1^). The as-obtained samples were denoted as xMg/NS, where x corresponds to the amount of magnesium (wt.%).

### 2.4. Further Modification with Silica

The materials were further modified with silica. The samples of 1 g xMg/NS were dispersed in the solution containing 1.07 g of CTAB, 71 mL of ethanol and 143 mL of water. The solution pH was adjusted to 11 using ammonia solution. Under vigorous stirring, TEOS (2.451 g) was added dropwise. After 4 h of mixing, the powder was separated by centrifugation (4500 rpm), washed with water and ethanol, dried at room temperature and calcined at 500 °C for 8 h (temperature ramp 1 °C·min^−1^). The as-obtained samples were denoted as NS/xMg/NS, where x corresponds to the amount of magnesium (wt.%).

### 2.5. Modification with Ruthenium

The obtained materials were impregnated with the same amount of ruthenium using aqueous solutions of ruthenium trichloride hydrate (concentration of Ru 4.656 mg·mL^−1^). After impregnation, the catalysts were dried overnight at 80 °C and calcined at 500 °C for 4 h (temperature ramp 1 °C·min^−1^). The as-obtained samples were denoted as Ru/NS/xMg/NS, where x corresponds to the amount of magnesium (wt.%).

### 2.6. Characterization

The samples were characterized with the use of advanced analytical techniques. Low temperature N_2_ adsorption/desorption measurements were performed using the Micromeritics ASAP 2020 instrument (Norcross, GA, USA). The sample (ca. 100 mg) was outgassed at 573 K under vacuum (<1.3 Pa) for 8 h. The pore volume and diameter were determined by BJH method.

Metals content (wt.%) were determined by ICP-OES, Spectro Blue TI (SPECTRO Analytical Instruments GmbH, Kleve, Germany). Prior to the analysis, samples were dissolved with the use of hydrofluoric acid and aqua regia.

XRD measurements were performed using a Bruker AXS D8 Advance diffractometer (Bruker, Karlsruhe, Germany) with Cu Kα radiation (λ = 0.154 nm) in the 2θ range of 25°–65° and at a step of 0.05°·s^−1^.

Scanning electron microscopy (SEM, FEI, Hillsboro, OR, USA) images were obtained with a FEI Quanta 250FEG microscope operating at 10 kV.

The X-ray Photoelectron Spectroscopy (XPS) measurements were carried out with a hemispherical analyser (SES R4000, Gammadata Scienta, Uppsala, Sweden). The unmonochromatized AlKα (1486.6 eV) X-ray source with the anode operating at 12 kV and 15 mA current emission was applied to generate core excitation. The energy resolution of the system, measured as a full width at half maximum (FWHM) for Ag 3d_5/2_ excitation line, was 0.9 eV (pass energy 100 eV). The spectrometer was calibrated according to ISO 15472:2001. The base pressure in the analysis chamber was about 1 × 10^−10^ mbar and about 3 × 10^−9^ mbar during the experiment. The analysed area of the sample was about 4 mm^2^ (5 mm × 0.8 mm). All spectra were collected at the pass energy of 100 eV (with 25 meV step). Intensities were estimated by calculating the integral of each peak (CasaXPS 2.3.23), after subtraction of the Shirley-type background, and fitting the experimental curve with a combination of Gaussian and Lorentzian lines of variable proportions (70:30). The results are charge-corrected (C-C bond, 285.0 eV) because samples were weakly conductive.

### 2.7. 2-Propanol Decomposition

The pelletized sample (50 mg) was placed in the fixed-bed reactor and activated in nitrogen flow (40 cm^3^·min^−1^) at 350 °C for 2 h. Next, the reactor was cooled down and 3 μL of 2-propanol were injected. Products were detected by a SRI 310 C gas chromatograph equipped with TCD and connected online with a microreactor. The reaction was carried out at 150, 200, 250 and 300 °C, however, only the results for 300 °C are shown as low conversions were observed at lower temperatures.

### 2.8. Hydrogenation of Levulinic Acid

The catalytic reaction was conducted in a 25 mL pressure batch Parr reactor. In a typical run 20 mL of 0.5 M aqueous levulinic acid solution was mixed with 50 mg of a catalyst. Prior to the reaction the catalyst was reduced in hydrogen flow (50 cm^3^·min^−1^) at 350 °C for 2 h. The reactor was flushed with helium and hydrogen three times, respectively. The reaction was carried out under 40 bar of hydrogen at 130 °C for 4 h with 600 rpm stirring. The only product (γ-valerolactone) was identified by GC-MS (Thermo Scientific, Waltham, Mass., USA) equipped with a 30 m DB-1 column. The conversion and yield were quantified with the use of a GC (Thermo Scientific, Waltham, MA, USA) equipped with 30 m DB-1 column and a FID detector.

## 3. Results and Discussion

### 3.1. Morphology and Textural Properties of the Solids

**Figure 1:**
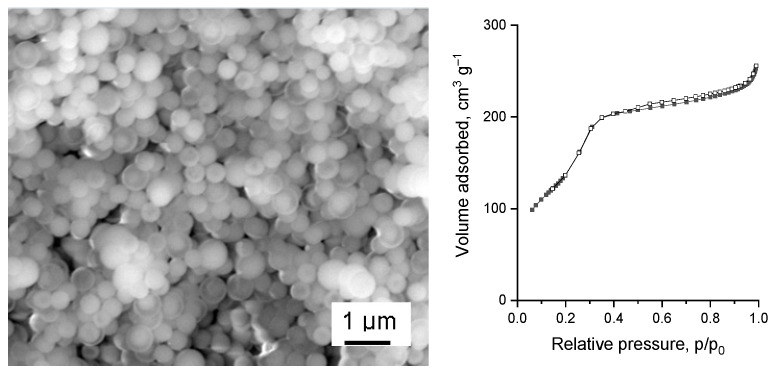
SEM image (**left**) and low temperature N_2_ adsorption/desorption isotherm (**right**) of porous silica nanospheres (NS).

Silica nanospheres were synthesized using a modified Stöber method [21]. The SEM image in Figure 1 confirmed the spherical morphology of the silica with the diameter of uniform spheres close to 650 nm. The silica was then etched with the use of Na_2_CO_3_ solution. The as obtained silica (NS) maintained its spherical morphology. The calculated etched spheres diameter was in the range 350–650 nm. The SEM image (Figure S1) of NS sample shows the effect of etching and generation of the voids in the silica particles.

The textural parameters of silica nanospheres were calculated on the basis of the low temperature nitrogen adsorption/desorption measurements. The material was found to have a relatively large surface area of 505 m^2^·g^−1^ (Table 1). The obtained isotherm type IV(b) with a hysteresis loop H4 indicated mesoporous character of the sample. The pore size distribution was uniform with a maximum at 3.3 nm. The as-obtained nanospheres also had a relatively large pore volume, i.e., 0.43 cm^3^·g^−1^.

The as-obtained support was further impregnated with three different loadings of magnesium, modified with porous silica, and impregnated with ruthenium (the same amount for each sample). As the references, the sample with only ruthenium (Ru/NS) and the sample with magnesium and ruthenium but with no further modification with silica (Ru/4Mg/NS) were prepared. Table 2 shows the concentrations of Mg and Ru in the samples estimated from the ICP and XPS measurements. The catalysts modified with silica (Ru/NS/3Mg/NS, Ru/NS/5Mg/NS, Ru/NS/6Mg/NS) contained 3, 5 and 6 wt.% of magnesium, respectively, according to ICP analysis. These results refer to the total metal concentration in the samples, whereas the XPS analysis reflects to the content of metal species located on the material surface. As expected, the modification of nanospheres containing Mg with silica, leads to a decrease in the concentration of magnesium on the materials surface. In contrast, for Ru/4Mg/NS sample, which was not enriched with silica, the concentration of magnesium was almost the same as estimated by the ICP and XPS techniques. The modification of samples with the same amount of ruthenium led to the same amount of this metal, ca. 0.35 wt.%, except for the sample Ru/NS. For this sample a little bit lower amount of Ru was detected. In contrast to the magnesium species, the concentration of ruthenium ones is much higher on the material surface that in the bulk, which is reasonable because ruthenium was incorporated into the materials at the last step of the modification.

The textural parameters of the catalysts determined at each step of the synthesis are collected in Table 1. The impregnation of silica with magnesium caused a decrease in the surface area, which is typical of silica modified especially with substances of basic nature. Further modification with silica resulted in the increase in the surface area of NS/3Mg/NS reaching 610 m^2^·g^−1^. This increase proves a successful modification of nanospheres with silica. However, the increase in the surface area was not observed for the material with the highest amount of magnesium, sample NS/6Mg/NS. This observation suggests a smaller efficiency of covering of 6Mg/NS sample with silica, which is also reflected in the smallest difference in the magnesium concentration measured by ICP and XPS for this sample. Interestingly, for NS/6Mg/NS, an increase in the total pore volume was observed, which indicates that some additional silica covers the 6Mg/NS sample. It should be concluded that the increase in the pore volume after the modification of xMg/NS materials with silica was a characteristic feature observed for all the materials. The last modification step, i.e., the incorporation of ruthenium, caused a decrease in the textural parameters for all catalysts. Nevertheless, the pore diameter of the samples oscillated around 3.3 nm throughout the modifications. Moreover, it should be noted that the highest surface area and total pore volume of the final materials were observed for Ru/NS/3Mg/NS. Apparently, generating porosity in post-modification with silica was successful as Ru/NS/3Mg/NS showed better textural parameters than Ru/4Mg/NS which had a similar content of metals.

**Figure 2:**
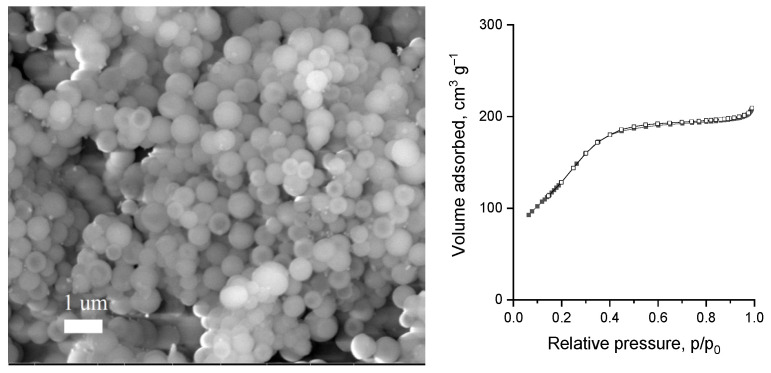
SEM image (**left**) and low temperature N_2_ adsorption/desorption isotherm (**right**) of Ru/NS/3Mg/NS.

It is also important to stress that the modification with metals and additional modification with silica did not alter the morphology of the catalysts as shown in the SEM images for Ru/NS/3Mg/NS (with corresponding isotherm of nitrogen adsorption/desorption) in Figure 2 and for other catalysts in Figure S2. Moreover, SEM-EDS analysis confirmed the uniform distribution of both metals in the catalyst as well as their content (Figure S3).

### 3.2. States of the Metals

**Figure 3:**
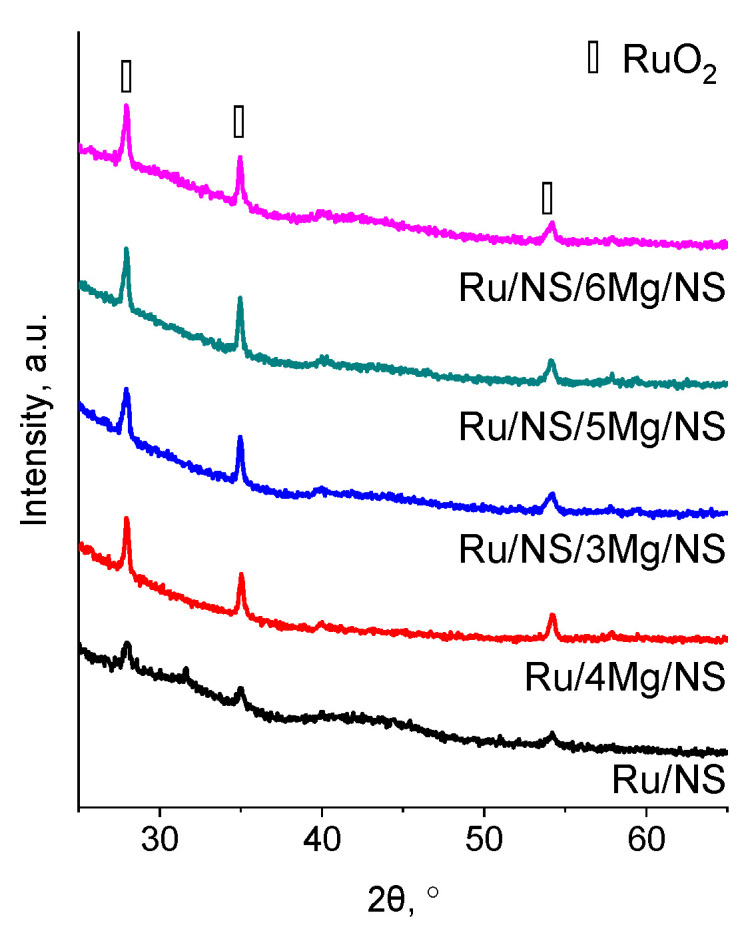
XRD patterns of the catalysts.

The states of the metals introduced into the catalysts were examined with the XRD technique. XRD patterns reveal the presence of reflections characteristic of crystalline ruthenium(IV) oxide (Figure 3). These reflections were observed for all ruthenium modified samples. A lower intensity of RuO_2_ reflexes in the XRD pattern of Ru/NS can be related to a slightly lower content of Ru in this sample as well as to smaller size of the crystals in it (see Table 2). The additional signal appearing uniquely in that pattern at 31.6° of 2θ is another signal of RuO_2_ as reported in literature [22]. In general, the RuO_2_ particles present on the catalysts’ surfaces were big in size, with their diameters ranging from 17 up to 28 nm. As mentioned above, relatively small diameters of RuO_2_ were observed for Ru/NS (16.8 nm). From among bimetallic samples, the smallest diameter of RuO_2_ was observed for Ru/NS/3Mg/NS (28.1 nm). Magnesium species were not detected possibly due to their amorphous character or very good dispersion.

XPS measurements provided an insight into the electronic states of the elements present on the surface of the catalysts obtained. The spectra and BE values are shown in Figure S4 and Table 3, respectively. The spectra collected in the Ru 3p_3/2_ region were deconvoluted into 2 pairs of peaks assigned to oxidized ruthenium species, the one at a lower BE corresponds to RuO_2_ and the other at a higher BE to Ru(OH)_3_ [23]. According to literature, the signals related to RuO_2_ are usually observed at lower values of BE, i.e., 462.5 eV [24,25], but taking into account the oxidizing conditions of the last stage of the synthesis as well as the results of XRD analyses, the signals in the range from 461.5 eV to 462.2 eV should be assigned to RuO_2_ species. The species assigned on the basis of the above-described deconvoluted peaks were confirmed by signals appearing in the Ru 3d region. Nevertheless, some difference in relative concentrations of both forms is noticed. This feature can be due to the less intense 3p peaks, as well as the difference in depths probed (~4 nm for Ru 3p compared to ~4.5 nm for Ru 3d).

For the samples modified with silica, the BE positions in the Ru 3p region are shifted towards lower values with increasing content of magnesium. The same tendency is observed for magnesium, i.e., BEs observed for the bands in Mg 1s region slightly decrease with increasing magnesium loading, i.e., for Ru/NS/3Mg/NS, Ru/NS/5Mg/NS, Ru/NS/6Mg/NS the maxima were at 1304.9 eV, 1304.4 eV and 1304.2 eV, respectively.

**Figure 4:**
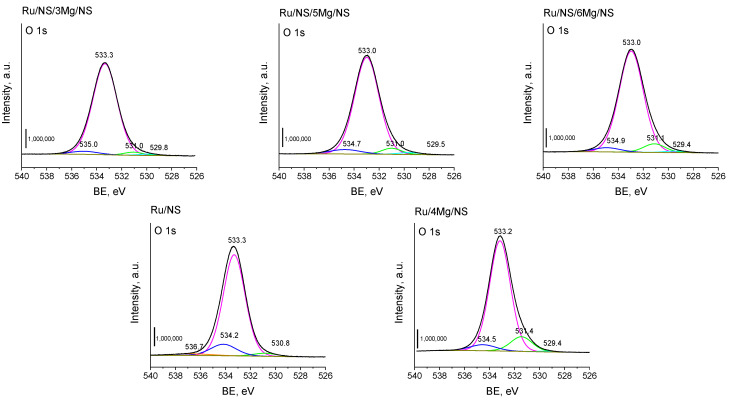
XPS spectra of the catalysts in the O 1s region.

A very important information concerning the modification efficiency of xMg/NS materials with silica can be inferred from the XPS spectra in the O 1s region (Figure 4). In this region a peak with BE 533.0–533.3 eV is assigned to oxygen in the silica lattice [26]. The contribution of this form of oxygen in Ru/NS and Ru/4Mg/NS is 83.8% and 82.5%, respectively. The modification of xMg/NS samples with TEOS in the presence of CTAB should result in enrichment of the silica on the material surface. Indeed, for the so-modified materials the contribution of lattice oxygen is in the range of 88.2% and 92.9% and has the highest value for Ru/NS/3Mg/NS. This is in line with the results of low temperature N_2_ adsorption measurements indicating for this sample the highest increase in the surface area after 3Mg/NS modification with TEOS. Moreover, it should be mentioned that the XPS spectra revealed the presence of another important oxygen species with BE in the range of 530.8–531.4 eV. This signal is assigned to metal oxides (e.g., RuO_2_ at 529.4 eV; MgO at 530.8–531.2 eV) [23,27]. For the Ru/NS/xMg/NS samples, the participation of these oxygen species increases with increasing amount of incorporated magnesium. This observation is reasonable taking into account quite similar amount of ruthenium on the surface of the materials obtained and the increase in the concentration of magnesium.

### 3.3. Catalytic Activity

The catalysts were tested in 2-propanol decomposition. In this reaction, depending on the character of the active site (acid, base or redox), the alcohol undergoes dehydration or dehydrogenation. According to Hattori [28], both reactions occur via elimination mechanism and are initiated by H^+^ abstraction. Depending on the location of the abstraction of H^+^, olefins or ketones are formed. Díez et al. [29] have tested magnesium oxide doped with various alkali metals and concluded that elimination leading to acetone production is favored reaction and takes place on medium-strength basic sites (Mg-O pairs). Olefins are formed over strong basic sites (O^2−^ ions derived from alkali metals). Some authors have suggested that acetone can also be produced over redox sites [30].

**Figure 5:**
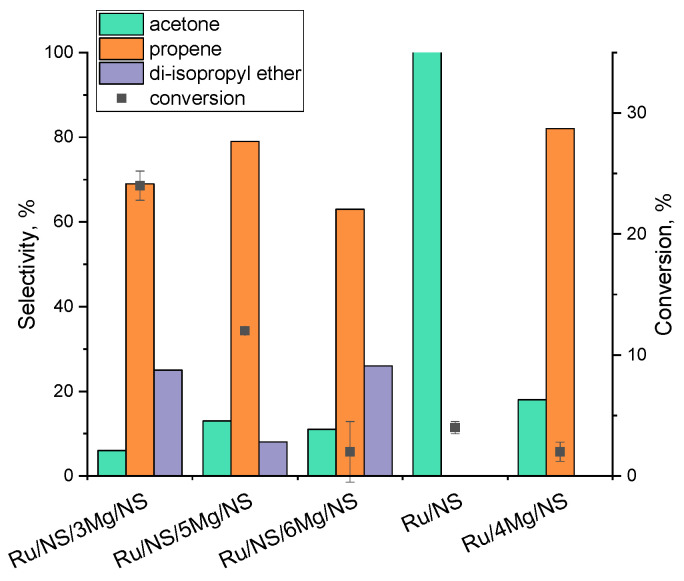
Catalytic results in the reaction of 2-propanol decomposition.

To characterize the active centres of the obtained materials, the samples were applied in the 2-propanol decomposition. The reaction was performed at 300 °C in nitrogen flow and the results obtained are shown in Figure 5. Ru/NS/3Mg/NS gave the highest conversion (24%) with selectivity to propene (69%) and di-isopropyl ether (25%). Twice lower conversion was observed for the catalyst with a higher loading of magnesium, Ru/NS/5Mg/NS, also with dominant selectivity to propene (79%). The remaining catalysts were almost inactive (2–4% of conversion), thus the comparison of their selectivity would be ambiguous, because it should be made for materials showing similar levels of conversion. Nevertheless, it can be noticed that the catalyst which did not contain magnesium, i.e., Ru/NS, was selective only to acetone, probably due to its redox properties. It should be also admitted that di-isopropyl ether was observed as a reaction product as well, and according to literature, it can be formed on the Lewis base and acid site pairs.

**Figure 6:**
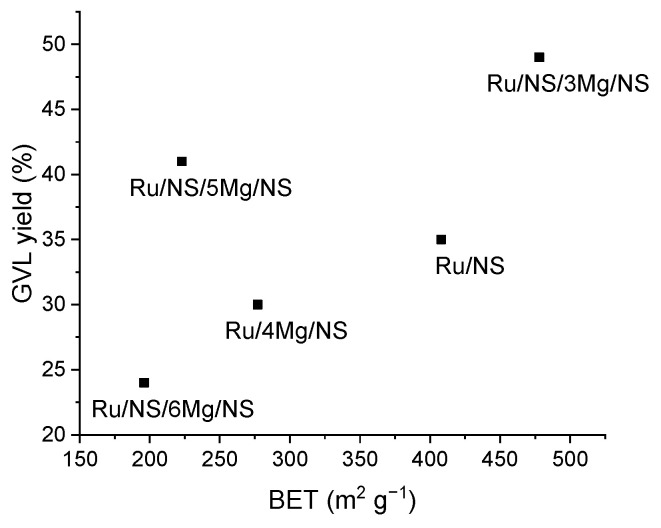
The relation between BET surface area of the catalyst and its yield to GVL.

The catalysts were also tested in the reaction of levulinic acid hydrogenation, and the relevant results are shown in Table 4. Firstly, it should be noted that the catalysts were only selective to γ-valerolactone (GVL). Despite the similar contents of ruthenium in the samples, they showed different catalytic performances. The highest yield of the target product, i.e., 49%, was obtained for the catalyst with the lowest loading of magnesium Ru/NS/3Mg/NS. The catalytic results also pointed to the positive role of xMg/NS modification with TEOS. It is clearly seen that for the catalysts with similar contents of ruthenium and magnesium, i.e., for Ru/NS/3Mg/NS and Ru/4Mg/NS, but without modification with silica, the conversion of levulinic acid was much lower (30%). Therefore, it should be noted that not only the chemical composition of catalyst plays an important role in the reaction. It seems that the texture of the catalysts is also crucial for the catalytic performance. As a result of modification of 3Mg/NS sample with TEOS, the final material containing ruthenium, i.e., Ru/NS/3Mg/NS, was characterized with the highest surface area and the largest pore volume from among the samples modified with silica (Table 1). It is well known that BET value can be a factor influencing the catalytic activity as it reflects the diffusion of reagents. Figure 6 illustrates the relation between the BET values of the catalysts and the yield of GVL. A well-seen general trend is that a large surface area favors GVL production. However, it is not the case for Ru/NS/5Mg/NS, as despite relatively low BET this catalyst was very active (41%). It should be mentioned that this material was also active in 2-propanol decomposition process.

To investigate the potential promoting effect of MgO dopant on the catalytic performance, the catalyst without magnesium (Ru/NS) was tested in the hydrogenation reaction (Table 4). Slightly lower content of ruthenium in Ru/NS (0.26 wt.%) in comparison to that in Ru/NS/3Mg/NS (0.32 wt.% of Ru) could have an impact on the lower conversion. On the other hand, the sample with slightly higher content of ruthenium and greater loading of magnesium, i.e., Ru/NS/5Mg/NS (0.35 wt.% of Ru), was less efficient in the reaction than Ru/NS/3Mg/NS. The same trend in activity was observed in 2-propanol decomposition (Figure 5). From among the catalysts doped with magnesium, the optimal loading with magnesium was found to be 3 wt.%. For this value of magnesium loading the modification with silica seems to be the most effective.

## 4. Conclusions

Silica nanospheres were modified with different amounts of magnesium species, covered with porous silica, and finally modified with ruthenium species. The results of low temperature N_2_ adsorption/desorption measurements performed after each step of the modification confirmed the successful covering of nanospheres with porous silica. This was also reflected in a lower concentration of magnesium species measured on the material surface than in the bulk, and higher concentration of oxygen species coming from silica lattice on the material surface, which was estimated from XPS measurement. The SEM images confirmed the spherical character of particles of the final samples. The highest efficiency of modification of nanospheres with porous silica was found for the material containing the smallest amount of magnesium species applied in this study, i.e., Ru/NS/3Mg/NS. This material showed also the higher activity in 2-propanol decomposition and levulinic acid hydrogenation to γ-valerolactone. For the latter process, a positive impact of surface area on catalytic activity was evidenced. Further detailed study on the stability of the proposed system and further optimization of the catalyst composition, e.g., the amount of metals, is required and will be the subject of the next work.

## Figures and Tables

**Table 1:** Textural properties of the catalysts.

	BET, m²·g^−1^	Pore Diameter, nm	Pore Volume, cm³·g^−1^
NS	505	3.3	0.43
Ru/NS	408	3.3	0.39
Ru/4Mg/NS	277	3.3	0.21
3Mg/NS	428	3.1	0.32
NS/3Mg/NS	610	3.6	0.45
Ru/NS/3Mg/NS	478	3.1	0.31
5Mg/NS	464	2.7	0.29
NS/5Mg/NS	494	3.3	0.38
Ru/NS/5Mg/NS	223	3.3	0.17
6Mg/NS	321	2.6	0.19
NS/6Mg/NS	287	3.3	0.24
Ru/NS/6Mg/NS	196	3.3	0.15

**Table 2:** Ruthenium content derived from ICP and XPS and ruthenium (IV) oxide particles’ size.

	Ru, wt.%		RuO_2_ Diameter, nm ^1^	Mg, wt.%	
	ICP	XPS		ICP	XPS
Ru/NS	0.26	2.2	16.7	-	-
Ru/4Mg/NS	0.35	2.9	28.1	3.6	3.3
Ru/NS/3Mg/NS	0.32	3.0	20.6	2.5	1.5
Ru/NS/5Mg/NS	0.35	3.0	26.9	4.9	3.0
Ru/NS/6Mg/NS	0.38	3.1	23.8	5.9	4.9

^1^ Calculated with Scherrer equation basing on the signal at 27.9° of 2θ from an XRD pattern.

**Table 3:** XPS BE values of the catalysts in the Ru 3p_3/2_, Ru 3d_5/2_ and Mg 1s region.

BE, eV	Ru/NS/3Mg/NS	Ru/NS/5Mg/NS	Ru/NS/6Mg/NS	Ru/NS	Ru/4Mg/NS
Ru 3p_3/2_	462.2 (50%)	461.8 (46%)	461.5 (53%)	462.0 (41%)	461.5 (53%)
	464.7 (50%)	464.1 (54%)	464.0 (47%)	464.4 (59%)	463.8 (60%)
Ru 3d_5/2_	281.0 (78%)	280.5 (40%)	280.4 (47%)	280.7 (64%)	280.4 (57%)
	282.5 (22%)	281.8 (60%)	282.0 (53%)	282.0 (36%)	282.0 (36%)
Mg 1s	1304.9	1304.4	1304.2	-	1304.5

**Table 4:** Activity of the catalysts in hydrogenation of levulinic acid. Reaction conditions: 20 mL of 0.5 M aqueous levulinic acid solution, 50 mg of the catalyst, 130 °C, 4 h, 40 bar of hydrogen.

Catalyst	GVL Yield,%
Ru/NS/3Mg/NS	49 ± 4.1
Ru/NS/5Mg/NS	41 ± 0.4
Ru/NS/6Mg/NS	24 ± 1.9
Ru/NS	35 ± 0.5
Ru/4Mg/NS	30 ± 0.3

## Data Availability

The data presented in this study are available on request from the corresponding author via e-mail: kalina.grzelak@amu.edu.pl (K.G.).

## Supplementary Materials

The following are available online at https://www.mdpi.com/article/10.3390/ma14237378/s1, Figure S1: SEM image of spherical nonporous silica, Figure S2: SEM images of the final materials Ru/NS/5Mg/NS and Ru/NS/6Mg/NS, Figure S3: The SEM-EDS analysis of Ru/NS/3Mg/NS: (a) SEM image, (b) Mg K scanning and (c) Ru L scanning, Figure S4: XPS spectra of the catalyst in Ru 3p and Ru 3d region.
