# Supplementary material for: Spherical Silica Modified with Magnesium and Ruthenium—Synthesis, Characterization and Catalytic Properties
Source: Materials (Basel). 2021 Dec 2;14(23):7378. doi: 10.3390/ma14237378 (PMC8658599; doi:10.3390/ma14237378)
Supplement: Supplementary file 1 [file materials-14-07378-s001.zip › materials-1475058-supplementary.pdf]

Supplementary Materials

Spherical Silica Modified with Magnesium and Ruthenium—Synthesis, Characterization and Catalytic Properties

Kalina Grzelak * and Maciej Trejda

Department of Heterogeneous Catalysis, Faculty of Chemistry, Adam Mickiewicz University, Uniwersytetu Poznańskiego 8, 61-614 Poznań, Poland; tmaciej@amu.edu.pl
* Correspondence: kalina.grzelak@amu.edu.pl

List of content:

Figure S1. SEM image of spherical nonporous silica.
Figure S2. SEM images of the final materials Ru/NS/5Mg/NS and Ru/NS/6Mg/NS.
Figure S3. The SEM-EDS analysis of Ru/NS/3Mg/NS: (a) SEM image, (b) Mg K scanning, (c) Ru L scanning.
Figure S4. XPS spectra of the catalyst in Ru 3p and Ru 3d region.

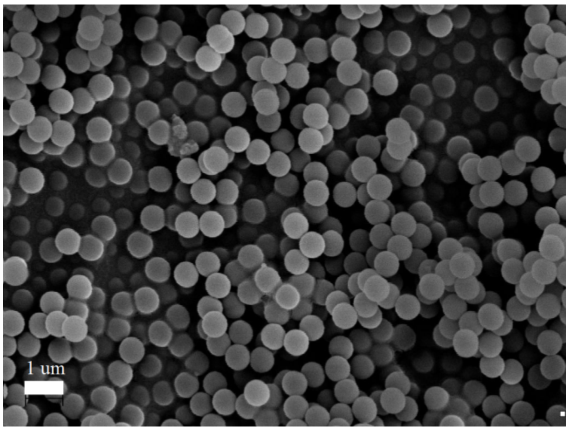

Figure S1. SEM image of spherical nonporous silica. (Scanning electron microscopy (SEM) image was carried out with a Quanta 250 FEG, FEI microscope operating at 10 kV.)

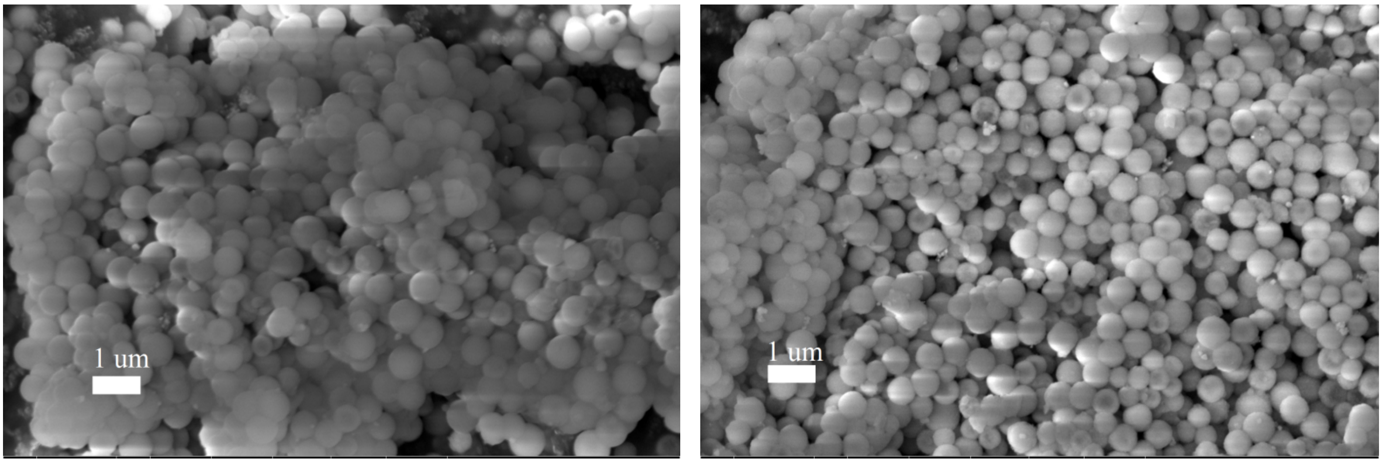

Ru/NS/5Mg/NS Ru/NS/6Mg/NS

Figure S2. SEM images of the final materials Ru/NS/5Mg/NS and Ru/NS/6Mg/NS.

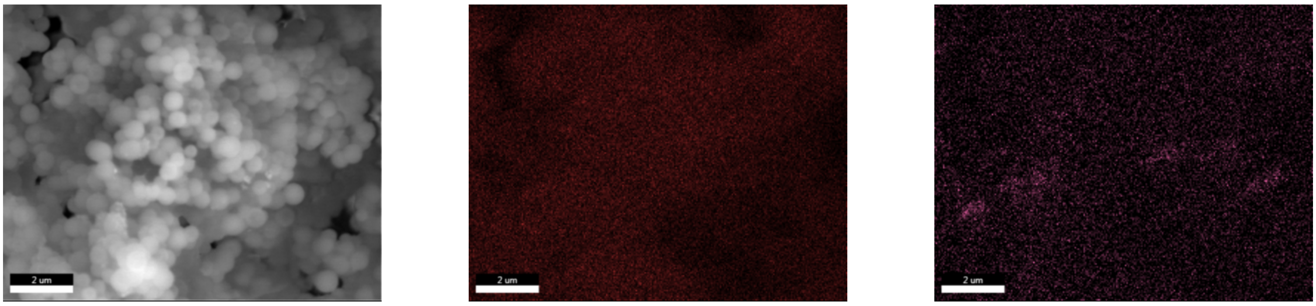

(a) (b) (c)

Figure S3. The SEM-EDS analysis of Ru/NS/3Mg/NS: (**a**) SEM image, (**b**) Mg K scanning, (**c**) Ru L scanning.

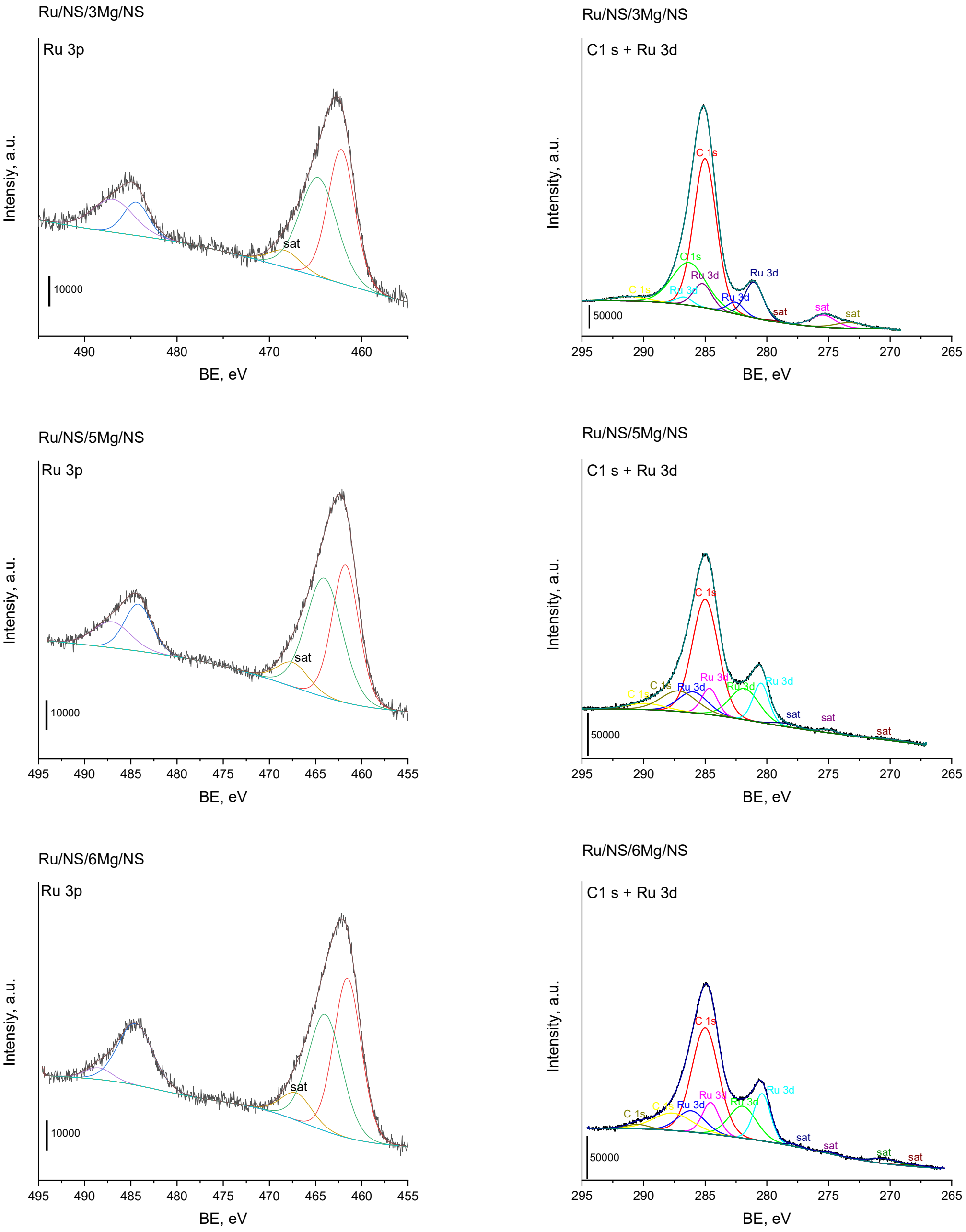

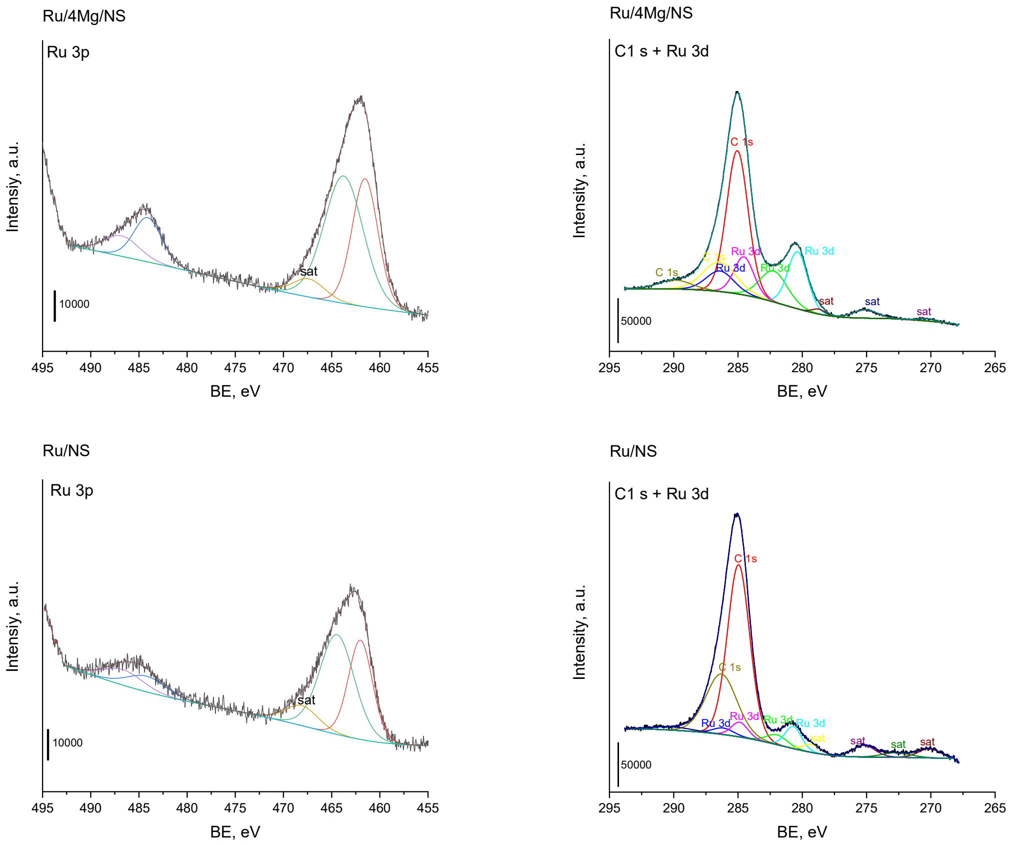

Figure S4. XPS spectra of the catalyst in Ru 3p and Ru 3d region.
